# Biosynthesis of the carbohydrate moieties of arabinogalactan proteins by membrane-bound β-glucuronosyltransferases from radish primary roots

**DOI:** 10.1007/s00425-013-1959-0

**Published:** 2013-09-22

**Authors:** Maya Endo, Toshihisa Kotake, Yoko Watanabe, Kazumasa Kimura, Yoichi Tsumuraya

**Affiliations:** 1 Division of Life Science, Graduate School of Science and Engineering, Saitama University, 255 Shimo-okubo, Sakura-ku, Saitama, 338-8570 Japan; 2 Yakult Central Institute for Microbiological Research, 1796 Yaho, Kunitachi, Tokyo 186-8650 Japan

**Keywords:** Cell wall, Glucuronic acid, Glycosyltransferase, *Raphanus*

## Abstract

A membrane fraction from etiolated 6-day-old primary radish roots (*Raphanus* *sativus* L. var *hortensis*) contained β-glucuronosyltransferases (GlcATs) involved in the synthesis of the carbohydrate moieties of arabinogalactan proteins (AGPs). The GlcATs transferred [^14^C]GlcA from UDP-[^14^C]GlcA on to β-(1 → 3)-galactan as an exogenous acceptor substrate, giving a specific activity of 50–150 pmol min^−1^ (mg protein)^−1^. The enzyme specimen also catalyzed the transfer of [^14^C]GlcA on to an enzymatically modified AGP from mature radish root. Analysis of the transfer products revealed that the transfer of [^14^C]GlcA occurred preferentially on to consecutive (1 → 3)-linked β-Gal chains as well as single branched β-(1 → 6)-Gal residues through β-(1 → 6) linkages, producing branched acidic side chains. The enzymes also transferred [^14^C]GlcA residues on to several oligosaccharides, such as β-(1 → 6)- and β-(1 → 3)-galactotrioses. A trisaccharide, α-l-Ara*f*-(1 → 3)-β-Gal-(1 → 6)-Gal, was a good acceptor, yielding a branched tetrasaccharide, α-l-Ara*f*-(1 → 3)[β-GlcA-(1 → 6)]-β-Gal-(1 → 6)-Gal. We report the first in vitro assay system for β-GlcATs involved in the AG synthesis as a step toward full characterization and cloning.

**Electronic supplementary material:**

The online version of this article (doi:10.1007/s00425-013-1959-0) contains supplementary material, which is available to authorized users.

## Introduction

Arabinogalactan proteins (AGPs) are a family of proteoglycans found in plant cell walls, plasma membranes, and the extracellular matrix. They are suspected to be important in signaling and communication in plants, and are implicated in diverse functions such as cell–cell recognition, cell fate, embryogenesis, and xylem development (Clarke et al. 1979; Fincher et al. 1983; Nothnagel 1997; Seifert and Roberts 2007). AGPs are characterized by large carbohydrate components rich in Gal and l-arabinose (l-Ara) and protein components (core proteins: generally <10 % of total weight) rich in hydroxyproline, Ser, Thr, Ala, and Gly. The carbohydrate moieties [arabinogalactan (AG)] of AGPs have a common basic structure of β-(1 → 3)-galactan backbones from which side chains of β-(1 → 6)-galactan branch off. The side chains are further substituted with l-Ara, glucuronic acid (GlcA), 4-*O*-methyl-glucuronic acid (4-Me-GlcA), and lesser amounts of other auxiliary sugars such as l-rhamnose and l-fucose. The uronosyl residues are found in nonreducing terminals attached to either β-(1 → 6)-galactan side chains or directly to β-(1 → 3)-galactan backbone chains as single residues through β-(1 → 6) linkages in radish AGPs (Tsumuraya et al. 1990).

It is known that the rate of turnover of the carbohydrate moieties of AGPs is very rapid (Gibeaut and Carpita 1991). We have observed that the sugar composition and structure of the peripheral region of the carbohydrate moieties of AGPs in radish plants are organ-specific and developmentally regulated depending on the growth stage of the plants (Tsumuraya et al. 1988). These characteristic features of AGPs imply that the biosynthesis and degradation of AGPs in plant tissues are coordinately regulated and interdependent processes. A degradation process proceeding by concerted action of several glycosidases such as β-galactosidase and α-l-arabinofuranosidase has been outlined in some plants (Kotake et al. 2005, 2006). On the other hand, the scenario for the biosynthesis process has not yet been described clearly. Since the carbohydrate moieties of AGPs are highly complex, there may be many glycosyltransferases for the synthesis of the glycan chains. These might include galactosyltransferases (GalTs) involved in the synthesis of the galactan [β-(1 → 3)(1 → 6)-galactan] chains as well as l-arabinosyl-, l-fucosyl-, and glucuronosyltransferases (Tan et al. 2012). However, our knowledge on the mechanisms and genes/enzymes responsible for the glycosylation of AGPs is limited. It is reasonable to assume that several different GalTs are required and act together in the synthesis of the galactan chains. Basu et al. (2013) and Oka et al. (2010) have detected and identified a GalT (Hyp:GalT) in *Arabidopsis* catalyzing the first step of the biosynthesis of the carbohydrate moieties by transferring Gal residues from UDP-Gal on to Hyp residues in the core proteins of AGPs. Subsequent chain elongation may proceed by addition of second Gal residues on to the first Gal residues by a different GalT (Gal:GalT). Qu et al. (2008) have identified a putative GalT, a member of the carbohydrate active enzyme (CAZy) glycosyltransferase (GT) family 31, responsible for synthesizing β-(1 → 3)-galactosyl linkages in AGPs. An *Arabidopsis* glycosyltransferase of GT31 (AtGALT31A) was found to possess GalT activity elongating β-(1 → 6)-galactan side chains of AGPs (Geshi et al. 2013). However, the properties of these putative GalTs are not yet fully characterized, and the mechanism and regulation of the formation of β-(1 → 3)-, β-(1 → 6)-galactosyl-, and β-(1 → 3)(1 → 6)-branching linkages remain elusive. Another example, indicating the variety of enzymes involved, has come from the study of Wu et al. (2010), who have identified and characterized two AGP-specific α-l-(1 → 2)-fucosyltransferases encoded by *AtFUT4* and *AtFUT6* genes in *Arabidopsis*.

However, definitive identification and proof of biochemical action of more individual genes and enzymes are still required to understand the complex process of AG synthesis. No β-glucuronosyltransferase (GlcAT) involved in the synthesis of AGs has been either characterized or cloned so far. We describe an in vitro assay for the activities of β-GlcATs that catalyze the incorporation of GlcA residues from UDP-GlcA into the carbohydrate moieties of AGPs in primary radish roots with various polysaccharides and oligosaccharides as acceptor substrates. We propose that different β-GlcATs catalyze the transfer of GlcA on to β-(1 → 3)-galactan backbones and β-(1 → 6)-galactan side chains of AGPs.

## Materials and methods

### Plant and chemical materials

Seeds of radish (*Raphanus sativus* L. var *hortensis* cv. Aokubi) were purchased from Tokita Seed and Plant (Saitama, Japan). Exo-β-(1 → 3)-galactanase (EC 3.2.1.145) from *Irpex lacteus* (Tsumuraya et al. 1990), endo-β-(1 → 6)-galactanase (EC 3.2.1.164) from *Trichoderma viride* (Okemoto et al. 2003), α-l-arabinofuranosidase (EC 3.2.1.55) from *Rhodotorula flava* (Uesaka et al. 1978), and β-glucuronidase (EC 3.2.1.31) from *Aspergillus niger* (Kuroyama et al. 2001) were prepared in our laboratories. Uridine 5′-diphospho-[^14^C]glucuronic acid (UDP-[^14^C]GlcA; 10.9 GBq mmol^−1^) was purchased from PerkinElmer Life Sciences Japan (Tokyo, Japan), while unlabeled UDP-GlcA was obtained from Sigma-Aldrich Japan (Tokyo, Japan). Triton X-100 was obtained from Wako Pure Chemical Ind. (Osaka, Japan). EGTA, Hepes, and Mes were from Dojindo Laboratories (Kumamoto, Japan).

β-(1 → 3)- and β-(1 → 6)-Galactobioses, and -trioses were prepared from larch wood AG by partial acid hydrolysis (Aspinall et al. 1958b). The β-(1 → 6)-galactotetraose was prepared from gum ghatti (Aspinall et al. 1958a). Chemically synthesized β-(1 → 6)-galactopentaose was donated by Dr. Miura, Gifu Pharmaceutical University, Japan, and Professor Inazu, Tokai University, Japan (Miura et al. 2004). β-GlcA-(1 → 6)-Gal and β-GlcA-(1 → 6)-β-Gal-(1 → 6)-Gal were prepared from acacia gum (Kuroyama et al. 2001). The α-l-Ara*f*-(1 → 3)-β-Gal-(1 → 6)-Gal (l-Ara·Gal·Gal) was prepared by enzymatic hydrolysis of the Smith degradation product of acacia gum with exo-β-(1 → 3)-galactanase (Tsumuraya et al. 1990). By repeated treatment of acacia gum, once to thrice Smith-degraded products were obtained (Sekimata et al. 1989; Tsumuraya et al. 1990), the last of which was essentially a linear β-(1 → 3)-galactan to which a small number (amounting to 7 %) of singleton β-Gal branches through β-(1 → 6)-linkages were attached. AGPs from radish roots (AGP-IV) and leaves (AGP R-II) were prepared as in Tsumuraya et al. (1984, 1988). Digestion of the AGPs with α-l-arabinofuranosidase provided enzymatically modified products. Further degradation of the modified root AGP with endo-β-(1 → 6)-galactanase yielded an additional product, which consisted of β-(1 → 3)-galactan backbone chains substituted with short neutral and acidic β-(1 → 6)-galactosyl side chains (Okemoto et al. 2003).

### Analytical methods

Protein content was determined by the method of Bradford (1976) using BSA as the standard. Total sugar content was determined by the phenol–sulfuric acid method (Dubois et al. 1956). Sugars were separated by paper chromatography on Whatman No. 1 or 3MM paper with 1-butanol:AcOH:water (5:2:3, by vol.) as the solvent system. Sugar spots on the chromatograms were visualized with alkaline AgNO_3_. Radioactive spots on paper chromatograms were detected by a Bio-Image Analyzer model BAS-1000 (Fuji Photofilm Co., Tokyo, Japan).

Oligosaccharides were labeled at their reducing terminals with *p*-aminobenzoic acid ethyl ester (ABEE) according to the method of Matsuura and Imaoka (1988). The ABEE-derivatized sugars were analyzed by HPLC with a Shimadzu LC-10AS fitted with a fluorescence detector, model RF-10A_XL_, and a column (250 mm long, 4.6 mm i.d.) of PALPAK Type N (Takara Bio Inc., Otsu, Japan). The column was eluted with acetonitrile:0.5 M acetic acid–triethylamine (pH 7.3) (90:10; v/v; solvent A) and acetonitrile:0.5 M acetic acid–triethylamine (pH 7.3) (50:50; v/v; solvent B). The column was eluted with a linear gradient of solvent B from 0 to 100 % over 50 min at a flow rate of 1 ml min^−1^ and at 40 °C. Fluorescence in the eluate was monitored at 305 nm (excitation) and 360 nm (emission). The apparent *M*_r_ of polysaccharides and oligosaccharides was estimated by size-exclusion HPLC with a refractive index detector (RID-10A) and tandem columns (each 300 mm long, 7.8 mm i.d.) of TSKgel G3000PW_XL_ and G2500PW_XL_ (Tosoh, Tokyo, Japan) as described previously (Kato et al. 2003). After passing through the columns and the detector, radioactive products were fractionated at 1-min intervals, and radioactivity (dpm) was counted with a liquid scintillation counter.

Matrix-assisted laser desorption/ionization time-of-flight mass spectrometry (MALDI-TOF/MS) was performed using a Kompact MALDI IV instrument (Shimadzu, Kyoto, Japan) as described previously (Kuroyama et al. 2001). The structure of the transfer product was examined by NMR. ^1^H-NMR and ^13^C-NMR spectra were recorded on a JEOL ECA-500 spectrometer (Tokyo, Japan) equipped with an inverse 5-mm gradient probe in D_2_O at 30 °C (Kotake et al. 2005). Chemical shifts were measured using acetone (*δ* 2.22 ppm for ^1^H-NMR and *δ* 30.5 ppm for ^13^C-NMR) as internal reference. The proton and carbon signals were assigned by the DQF-COSY, 1D-TOCSY, DEPT135, HC-HSQC, and HC-HMBC spectroscopy experiments. The signals for glycosylated carbons were confirmed by their markedly higher *δ* values compared with those for the corresponding non-glycosylated ones. Gas–liquid chromatography (GLC) of sugars as alditol acetates was performed with a Shimadzu gas chromatograph GC-6A fitted with a column (0.28 mm × 50 m) of Silar 10C, according to the method of Albersheim et al. (1967). The carboxyl groups of GlcA residues in permethylated oligosaccharides were reduced with LiAlH4 (Lewis et al. 1963), and the resulting methylated derivatives of Glc were analyzed by GLC.

### Assays of β-GlcAT

A microsomal fraction from 6-day-old primary roots of radish was prepared according to the method of Misawa et al. (1996).

#### Assay with radiolabeled UDP-GlcA

The activities of β-GlcATs were assayed by the procedure used by Kato et al. (2003), which was adopted for radish β-GalT. A standard reaction mixture (total volume 30 μl) contained 2.0 mM UDP-[^14^C]GlcA (0.069 nmol radiolabeled compound equivalent to 0.74 kBq, supplemented with 60 nmol of unlabeled compound), β-(1 → 3)-galactan (5 mg ml^−1^), 30 mM MnCl_2_, 0.75 % (w/v) Triton X-100, 160 mM sucrose, 50 mM galactono-(1 → 4)-lactone, 20 mM NaF, 0.4 mM DTT, 40 mM Mes-KOH buffer (pH 6.0), and the microsomal fraction (protein content 30–50 μg). Galactono-(1 → 4)-lactone was added to inhibit microsomal β-galactosidase(s) (Kato et al. 2003), which might degrade β-(1 → 3)-galactan, AGP, and β-galactooligosaccharides provided as acceptor substrates in the reaction mixtures. The mixture was incubated at 25 °C for 60–120 min. After the reaction was terminated with 0.3 M acetic acid (80 μl) containing 20 mM EGTA, aliquots (100 μl) were subjected to paper chromatography using 95 % ethanol:1 M ammonium acetate (2:1, v/v) as the solvent. The radiolabeled products, which were immobilized on the base line of chromatograms, were cut off, sonicated in water (2 ml), and radioactivity was counted with a liquid scintillation counter (Ishikawa et al. 2000). Incorporation of [^14^C]GlcA into other polysaccharides was measured similarly. Parallel assays were done under the standard assay conditions without addition of acceptors for estimation of β-GlcAT activity to endogenous acceptors. Activities with or without acceptors were corrected by respective time-zero controls. Unless otherwise noted, the data given are the net amounts of [^14^C]GlcA incorporated into acceptors obtained by subtracting the amounts to endogenous acceptors from the total amounts in the presence of exogenous acceptors.

Incorporation of [^14^C]GlcA into oligosaccharide acceptors was measured under the same conditions as above except for acceptor oligosaccharides (4 mM). The subsequent procedure followed the method of Misawa et al. (1996). The reaction was stopped by addition of cold water (1 ml) and radiolabeled products were applied to a small column (~1 ml) of DEAE-cellulose (Serva, Heidelberg, Germany). After washing with cold water (3 ml), the adsorbed radiolabeled transfer products were eluted in steps with 0.02 M NaHCO_3_ followed by 0.1 M NaHCO_3_ (each 4 ml) at room temperature. Each 1 ml of the eluate was collected and radioactivity in 0.02 M fractions was counted. Enzyme activity was corrected by controls incubated and treated in a similar manner without addition of acceptors.

Enzyme assays were done, at least, in duplicate and mean values were recorded.

#### Assay with non-radiolabeled UDP-GlcA

The action of β-GlcATs toward oligosaccharide acceptors was also examined by a fluorescence-labeling method. The reaction mixture (30 μl) consisted of 2.0 mM UDP-GlcA, 4 mM oligosaccharide acceptors, the microsomal fraction, and the same concentrations of other components as the standard assay mixture without addition of sucrose and galactono-(1 → 4)-lactone. The reaction was conducted for a prolonged time (4 h) and terminated by heating in a boiling water bath for 1 min. The reaction products were derivatized with ABEE, filtered through a membrane filter (Chromatodisk 4A; Kurabo, Osaka, Japan), and subjected to HPLC analysis. The amount of product was expressed as the percentage of GlcA transferred from UDP-GlcA to acceptor oligosaccharides.

### Analysis of transfer products

#### Product from polysaccharide acceptors

We found that the commercial UDP-[^14^C]GlcA specimen contained a small amount of impurity that interfered with the analysis of the products formed by transfer of [^14^C]GlcA. Therefore, the specimen was purified by preparative paper chromatography using 95 % ethanol:1 M ammonium acetate (2:1, v/v) as the solvent as described above. The areas corresponding to UDP-[^14^C]GlcA were cut off, extracted with hot water, centrifuged, concentrated, and used for the following experiments. A large-scale reaction mixture (150 μl, membranous protein 770 μg) containing 15 times the amounts of UDP-[^14^C]GlcA (11.1 kBq) and β-(1 → 3)-galactan (5 mg ml^−1^) and the same concentrations of other components as in the standard assay mixture was incubated at 25 °C for 24 h. After heating the reaction mixture, the transfer product was isolated by paper chromatography as above. The radiolabeled products immobilized on the base line of chromatograms were extracted with hot water, centrifuged, and concentrated to dryness. The amount of [^14^C]GlcA incorporated into the transfer product was 5.6 % of initial UDP-[^14^C]GlcA. Another transfer product was obtained in a similar manner as above using α-l-arabinofuranosidase followed by endo-β-(1 → 6)-galactanase-digested root AGP as the acceptor, where [^14^C]GlcA transfer was 1.6 % of initial UDP-[^14^C] GlcA.

A portion (3,000–7,000 dpm) of each extract was digested with either exo-β-(1 → 3)-galactanase (17 milliunits) or β-glucuronidase (9 milliunits) or both enzymes in a reaction mixture (20 μl) containing 12 mM sodium acetate buffer (pH 4.6), at 37 °C for 24 h. After heating the reaction mixture, a part of the hydrolysate was separated by paper chromatography and analyzed for radioactivity with a Bio-Image Analyzer. Another part was subjected to size-exclusion HPLC and analyzed for radioactivity with a liquid scintillation counter.

#### Products from oligosaccharide acceptors

A large reaction mixture (3 or 1.5 ml) containing 10 or 6 mg membrane protein, respectively, 2 mM UDP-GlcA, and 4 mM β-(1 → 6)- or β-(1 → 3)-galactotriose, or l-Ara·Gal·Gal as acceptor, and the same concentrations of other components as the standard assay mixture without sucrose and galactono-(1 → 4)-lactone was incubated at 25 °C for 24 h. After heat-inactivation of the reaction, the transfer products in the reaction mixture were derivatized with ABEE and partially purified on a column (92 cm long, 1.6 cm i.d.) of Sephadex G-15 (GE Healthcare Bio-Sciences, Tokyo, Japan) eluted with 10 mM ammonium bicarbonate. The fractions containing the transfer products were concentrated by evaporation and applied to a Presep-C C-18 (ODS) cartridge (Wako Pure Chemical Ind.). The cartridge was washed with 0.1 M acetic acid, and the products were eluted with 20 % acetonitrile and concentrated. The products were then purified by HPLC. The products recovered were treated with the cartridge once more and dried.

The transfer products were analyzed for their structure by MALDI-TOF/MS, NMR, methylation analysis, and enzymatic hydrolysis with β-glucuronidase or α-l-arabinofuranosidase. The enzymatic hydrolysis products were analyzed by HPLC.

## Results

**Fig. 1:**
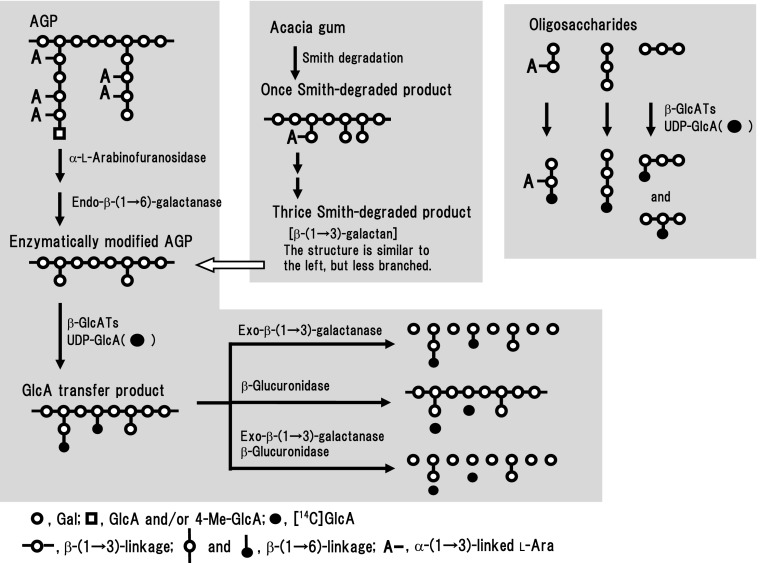
Schematic overview of the transfer of GlcA from UDP-GlcA on to β-(1 → 3)-galactan, enzymatically modified AGP, and oligosaccharides by the action of radish β-GlcATs. Once to thrice Smith-degraded products were prepared from acacia gum: thrice Smith-degraded product was essentially β-(1 → 3)-galactan containing a small amount (7 %) of single Gal residues attached through β-(1 → 6) linkages. The enzymatically modified AGP was prepared by successive digestion of radish root AGP with α-l-arabinofuranosidase and endo-β-(1 → 6)-galactanase. More detailed structural models are shown in a reference (Kitazawa et al. 2013 and the supplemental data for it). The [^14^C]GlcA transfer products produced by the enzymatic action were analyzed by enzymatic hydrolysis with exo-β-(1 → 3)-galactanase, β-glucuronidase, and successive digestion with exo-β-(1 → 3)-galactanase and β-glucuronidase (see Fig. 4)

A schematic overview of this study is shown in Fig. 1, which includes structural models of β-(1 → 3)-galactan and radish AG, and of the oligosaccharides used in this study as efficient acceptor substrates. The figure also shows the GlcA transfer action of radish β-GlcATs on to these acceptors, and the analytical diagram of [^14^C]GlcA transfer products by enzymatic hydrolyses (see Fig. 4).

### β-GlcAT activities in microsomes

**Fig. 2:**
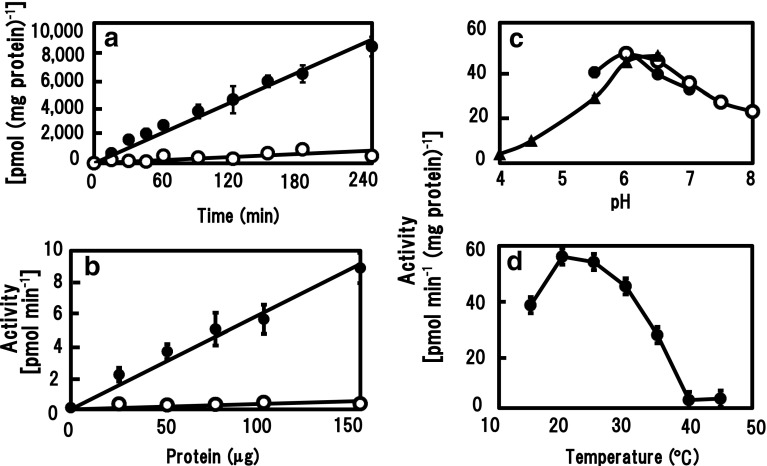
Activity of β-GlcATs in microsomes from radish primary roots. **a** The microsomes (protein, 50 μg) were incubated under standard assay conditions with β-(1 → 3)-galactan as exogenous acceptor (*closed circles*) and with endogenous acceptors (*open circles*) for varying times up to 240 min. After 240 min, the amount of [^14^C]GlcA transferred to β-(1 → 3)-galactan was about 0.6 % of initial UDP-[^14^C]GlcA. The data are the mean + SE of quadruple experiments. **b** Relationship between enzyme activity and protein concentration. Various amounts of microsomal protein were incubated under standard conditions for 60 min. **c** Effects of pH on enzyme activity. Activity–pH curves result from experiments with buffers (80 mM) having different pH values under standard conditions for 120 min. *Closed circles* Mes–KOH, *open circles* Hepes–KOH, *closed triangles* acetate. **d** Activity–temperature curves result from incubations at different temperatures under standard assay conditions for 120 min. Note that the activity plotted for β-(1 → 3)-galactan includes that for endogenous acceptors in **a** and **b**

The activities of β-GlcATs were assayed by incubating a microsomal fraction from 6-day-old primary roots of radish under standard assay conditions with β-(1 → 3)-galactan as the acceptor substrate. The transfer products were separated by paper chromatography from unreactive UDP-[^14^C]GlcA and its degraded product, and their radioactivity was measured. Enzyme product with β-(1 → 3)-galactan increased proportionally up to 240 min (Fig. 2a), and activity increased proportionally to the amount of protein up to 150 μg under an incubation time of 60 min (Fig. 2b). Activity for endogenous acceptors increased proportionally, but was low (about one-tenth) in comparison with that of the standard reaction. Control experiments were done under standard conditions either without microsomes or with heat-denatured membrane proteins instead of intact microsomes. These gave no detectable activity. The specific activity (after subtraction of activity toward endogenous acceptors) of radish β-GlcATs was usually in a range of 50–150 pmol min^−1^ (mg protein)^−1^ [i.e., 8–25 pkat (mg protein)^−1^], depending on the microsomal preparations. Actual activity to endogenous acceptors was in a range of 5–15 pmol min^−1^ (mg protein)^−1^. The total activity differed depending on radish organs after 6-day cultivation: it was lower in cotyledons (14 %) and hypocotyls (36 %), where the specific activity of root GlcAT is taken as 100 %.

The specific activity of β-glucuronidase(s) in the microsomes was assayed in a mixture containing 1 mM *p*-nitrophenyl-β-glucuronide, 50 mM acetate buffer (pH 4.6), and the microsomal fraction at 25 °C for 4 h (Kuroyama et al. 2001). The hydrolysis activity was 58 pmol min^−1^ (mg protein)^−1^, almost the same magnitude as that of β-GlcATs. The enzyme seems to degrade, partially, the transfer products formed by the action of β-GlcATs, thus leading to an underestimation of the activities of β-GlcATs.

### Properties of β-GlcATs

The effect of pH on the activity of the β-GlcATs was examined with three kinds of buffers of various pH at a final concentration of 80 mM under standard assay conditions with β-(1 → 3)-galactan as acceptor. The optimum pH for β-GlcAT action was around 6.0 (Fig. 2c). Enzyme activity was optimal at 20–25 °C (Fig. 2d).

The effect of addition of various detergents on β-GlcAT activity was examined at 0.5 % (w/v) final concentration under standard assay conditions. The following data were obtained, where the number in parentheses represents the relative activity, taking that obtained without detergent as unity: none (1), Triton X-100 (3.7), digitonin (3.6), Zwittergent 3–16 (2.2), *n*-octyl-β-thioglucoside (1.5). The effect of various concentrations (0.05–1.5 %, w/v) of Triton X-100 on enzyme activity was measured. The activity increased with the concentration of Triton X-100 but reached a plateau at 0.75 % with fivefold higher activity than without the detergent. It seems likely that the inclusion of Triton X-100 in reaction mixtures solubilizes membrane-bound β-GlcAT proteins or loosens them to facilitate access to substrates. We tested for this possibility by mixing the microsomes (0.6 ml; 1.6 mg protein) with the standard assay mixture except for substrates. The mixture was kept at 0 °C for 30 min, and centrifuged at 100,000*g* for 1 h. The proportion of total activity recovered in the supernatant and the pellet was 50 and 30 % of initial activity, indicating that about one-third of β-GlcAT protein remains in a membrane-bound form in reaction mixtures.

The effect of various cations on β-GlcAT activity was examined by incubation at 5 mM final concentration. The following results were obtained, taking that obtained without cation as unity: none (1), Mn^2+^ (2.8), Mg^2+^ (2.3), Zn^2+^ (1.6), Co^2+^ (1.5), Ca^2+^ (1.2). Apparently, Mn^2+^ and Mg^2+^ enhanced activity. The effects of various concentrations (1–70 mM) of Mn^2+^ were examined. Activity increased with increasing concentration of Mn^2+^, reaching a maximum at 30–40 mM concentration (4 times that without the ion), then gradually declined at higher concentrations.

**Fig. 3:**
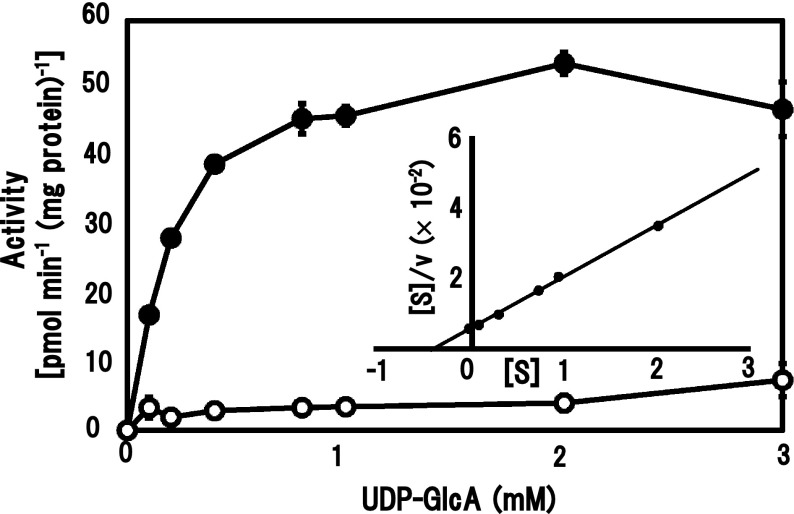
Effect of UDP-GlcA concentration on β-GlcAT activities. Enzyme activity was measured under standard assay conditions by incubation of the membranes (protein, 50 μg) at various concentrations of UDP-[^14^C]GlcA for 120 min. The *inset* represents the Hanes–Woolf plot for the data. Refer Fig. 2 for the symbols

The effect of the UDP-[^14^C]GlcA concentration (0–3 mM) on β-GlcAT activity was examined. Apparent *K*_m_ and *V*_max_ values for UDP-GlcA, calculated according to the Hanes–Woolf plot, were 0.26 mM and 60 pmol min^−1^ (mg protein)^−1^, respectively (Fig. 3). Kinetic analysis for the effect of concentrations (0–12 mg ml^−1^) of β-(1 → 3)-galactan did not give a saturated curve of reaction velocity and showed almost linearly increasing activity, 150 pmol min^−1^ (mg protein)^−1^ at 12 mg ml^−1^. Hence, significant kinetic parameters were not obtained.

The following sugars inhibited β-GlcAT activities at high (50 mM) concentration, where that obtained without sugars is taken as standard: none (1), galacturonic acid (0.003), glucono-(1 → 5)-lactone (0.02), glucuronic acid 1-phosphate (0.54). However, these sugars did not affect the activity as much at low (10 mM) concentration. GlcA did not affect activity even at 50 mM concentration. Sucrose (160 mM) and galactono-(1 → 4)-lactone (50 mM), which were included in the standard assay mixture, neither enhanced nor inhibited enzyme activities.

### Substrate specificity

Various AGPs, their enzymatically modified derivatives, and polysaccharides were examined for efficiency as acceptor substrates (Table 1). Among these AGPs and polysaccharides, β-(1 → 3)-galactan used as an acceptor for the standard enzyme assay showed the highest activity. Native acacia gum was a poor substrate, but enzyme activity tended to increase with the proportion of (1 → 3)-linked β-Gal residues, which was increased by successive Smith degradations. However, once Smith-degraded product was a better acceptor than twice degraded product. The once degraded product has a lower proportion of (1 → 3)-linked β-Gal residues than twice Smith-degraded product, but has α-l-Ara*f* residues (21 mol %) attached to β-(1 → 6)-galactosyl side chains through (1 → 3) linkages (Tsumuraya et al. 1990). We suggest that the α-l-Ara*f* residues facilitate the transfer of GlcA residues from UDP-GlcA, causing the enhancement of β-GlcAT activity. This is consistent with the result obtained when l-Ara-containing galactooligosaccharide (l-Ara·Gal·Gal) was used as an acceptor (see below).

**Table 1:** Effect of various AGPs and polysaccharides on the activity of ß-GlcATs

Acceptor^a^	Enzyme activity (%)^b^
Acacia gum	
Native	25
Once Smith-degraded product	63
Twice Smith-degraded product	36
Thrice Smith-degraded product	100
Radish root AGP	
Native	26
α-l-Arabinofuranosidase treated	50
α-l-Arabinofuranosidase + endo-β-(1 → 6)-galactanase treated	91
α-l-Arabinofuranosidase + exo-β-(1 → 3)-galactanase treated	9
Radish leaf AGP	
Native	8
α-l-Arabinofuranosidase treated	12
Larch wood AG	8
Soybean arabinan-galactan	0
Oat-spelt xylan	60

^a^Concentrations of acceptors were 5 mg ml^−1^

^b^Activity expressed relative to that for thrice Smith-degraded product. In other words, total activity toward almost unadorned β-(1 → 3)-galactan, 59 pmol min^−1^ (mg protein)^−1^, was taken as 100 %. The mixtures were incubated at 25 °C for 120 min. Activity of the enzyme preparation, 1 pmol min^−1^ (mg protein)^−1^, toward endogenous acceptors is subtracted

Native root AGP was a poor acceptor but turned into a better substrate when Gal residues were exposed by enzymatic removal of α-l-Ara*f* residues (content in the AGP, 24 mol %; Tsumuraya et al. 1988) that are attached at *O*-3 of nonreducing terminal and internal β-(1 → 6)-galactosyl side chains ranging from single to, at least, 20 Gal residues with or without caps of single 4-Me-GlcA residues at nonreducing terminals (Tsumuraya et al. 1990; Haque et al. 2005). Subsequent digestion of the α-l-arabinofuranosidase-modified AGP by endo-β-(1 → 6)-galactanase removes such β-(1 → 6)-galactosyl side chains and leaves short stubs [mostly a single unit long, some of which are substituted with 4-Me-GlcA residues (Okemoto et al. 2003)]. Consistent with the results obtained for the series of chemically modified acacia gum, α-l-arabinofuranosidase-modified root AGP showed enhanced efficiency, half the enzyme activity of β-(1 → 3)-galactan. Similar results were obtained for the case of leaf AGP. The α-l-arabinofuranosidase- and β-(1 → 6)-galactanase-modified root AGP served as a very effective acceptor, with activity amounting to as much as 90 % of that of β-(1 → 3)-galactan. These results suggest that radish primary root GlcATs prefer long consecutive (1 → 3)-linked β-Gal residues as acceptor substrates for GlcA transfer. On the other hand, when the α-l-arabinofuranosidase-modified root AGP was further digested with exo-β-(1 → 3)-galactanase, the resulting AGP showed lower efficiency than the native AGP. This exo-β-(1 → 3)-galactanase-modified AGP has lost more than 90 % of the sugar constituents of the native form and represents the core portion of the AGP with only a small portion of (1 → 3)-linked β-Gal residues remaining (Tsumuraya et al. 1990). Larch wood AG and soybean arabinan–galactan (Sekimata et al. 1989) were unreactive, but oat-spelt xylan (Sigma-Aldrich, Japan) served as a good acceptor. We confirmed that the xylan specimen was free from AGPs by gel diffusion assay with β-galactosyl Yariv reagent (Biosupplies, Bundoora, Australia), a dye specific to AGPs (Nothnagel 1997). Our result suggests that the xylan acted as an acceptor for α-GlcAT(s) involved in the synthesis of glucuronoxylan.

Table 2 summarizes the activities of β-GlcATs with Gal and various galactooligosaccharides differing in type of linkage and chain length. Clearly, Gal was a poor acceptor substrate, and β-(1 → 6)- and β-(1 → 3)-galactobioses were also seen to be unreactive when the amount of [^14^C]GlcA detected in the control reaction incubated without acceptors was taken into account. The activity, however, increased with the increasing chain length of both (1 → 6)- and β-(1 → 3)-linked oligosaccharides as evidenced by the high activity observed for β-(1 → 6)-galactooligosaccharides with degree of polymerization (DP) 3–5 and β-(1 → 3)-galactotriose. As observed for polymer acceptors, radish β-GlcATs seem to prefer consecutive (1 → 3)-linked β-Gal residues. They also appear to prefer consecutive (1 → 6)-linked β-Gal residues. This contrasts with the results observed for polymer acceptors, in which native root AGP and its α-l-arabinofuranosidase-modified derivative served as less efficient acceptors than α-l-arabinofuranosidase- and β-(1 → 6)-galactanase-modified AGP with shortened β-(1 → 6)-galactosyl side chains. In addition, we observed that an l-Ara-containing galactooligosaccharide, l-Ara·Gal·Gal, served as a better acceptor than β-(1 → 6)-galactobiose, suggesting that the l-Ara*f* residues facilitate the transfer of GlcA residues from UDP-GlcA catalyzed by β-GlcATs.

**Table 2:** Substrate specificity of radish ß-GlcATs toward various oligosaccharide acceptors

Acceptor	Enzyme activity [pmol min^−1^ (mg protein)^−1^]^a^
Control (endogenous acceptors)	45
Gal	49
β-(1 → 6)-galactobiose	43
β-(1 → 6)-galactotriose	89
β-(1 → 6)-galactotetraose	84
β-(1 → 6)-galactopentaose	104
β-(1 → 3)-galactobiose	58
β-(1 → 3)-galactotriose	90
α-l-Ara*f*-(1 → 3)-β-Gal-(1 → 6)-Gal (l-Ara·Gal·Gal)	93

^a^Activity expressed as specific activity calculated based on the amounts of [^14^C]GlcA transfer products eluted from ion-exchange chromatography of the reaction mixtures. The reaction was carried out at 25 °C for 120 min. The high control value observed for incubation without acceptors may derive from [^14^C]GlcA transferred to endogenous acceptors as well as free [^14^C]GlcA due to partial degradation of UDP-[^14^C]GlcA during incubation

### Analysis of transfer products obtained with β-(1 → 3)-galactan and a modified AGP

**Fig. 4:**
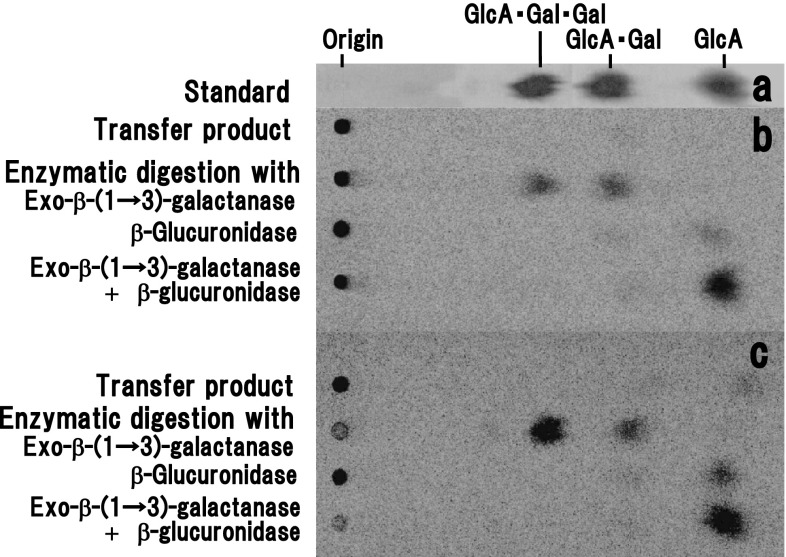
Analyses of the enzymatic hydrolysates of the [^14^C]GlcA transfer products by paper chromatography. **a** Standard sugars: GlcA, β-GlcA-(1 → 6)-Gal, and β-GlcA-(1 → 6)-β-Gal-(1 → 6)-Gal (*from right to left*). These sugars were detected by the alkaline AgNO_3_ method. The transfer product formed from β-(1 → 3)-galactan (**b**) and a root AGP digested successively with α-l-arabinofuranosidase and endo-β-(1 → 6)-galactanase (**c**) as exogenous acceptors were digested, respectively, with enzymes, and the pattern of radioactivity was analyzed by Bio-Image Analyzer. All intact transfer products and their digestion products with exo-β-(1 → 3)-galactanase, β-glucuronidase, and successive digestion with exo-β-(1 → 3)-galactanase and β-glucuronidase are shown

The products of [^14^C]GlcA transfer on to β-(1 → 3)-galactan and α-l-arabinofuranosidase- and β-(1 → 6)-galactanase-modified AGP by the action of radish β-GlcATs were characterized by enzymatic analysis. The transfer products formed from β-(1 → 3)-galactan were digested with either exo-β-(1 → 3)-galactanase, or β-glucuronidase, or exo-β-(1 → 3)-galactanase followed by β-glucuronidase (Fig. 4). Separation of the hydrolysis products on paper chromatography showed main radiolabeled sugars corresponding to β-GlcA-(1 → 6)-Gal and β-GlcA-(1 → 6)-β-Gal-(1 → 6)-Gal after exo-β-(1 → 3)-galactanase treatment. The oligosaccharides gave [^14^C]GlcA as the sole radioactive sugar after subsequent digestion with β-glucuronidase. This was also the case for the enzymatically modified AGP (Fig. 4c). Based on the substrate specificity of the enzymes (Tsumuraya et al. 1990; Kuroyama et al. 2001), radiolabeled β-GlcA-(1 → 6)-Gal arises from single units of [^14^C]GlcA transferred on to nonreducing terminals and/or internal Gal residues along β-(1 → 3)-galactan chains through β-(1 → 6) linkages. We suggest that radiolabeled β-GlcA-(1 → 6)-β-Gal-(1 → 6)-Gal derived from [^14^C]GlcA transferred on to single-unit β-(1 → 6)-galactosyl branches attached to β-(1 → 3)-galactan and α-l-arabinofuranosidase- and β-(1 → 6)-galactanase-modified AGP through β-(1 → 6) linkages, as schematically depicted in Fig. 1.

**Fig. 5:**
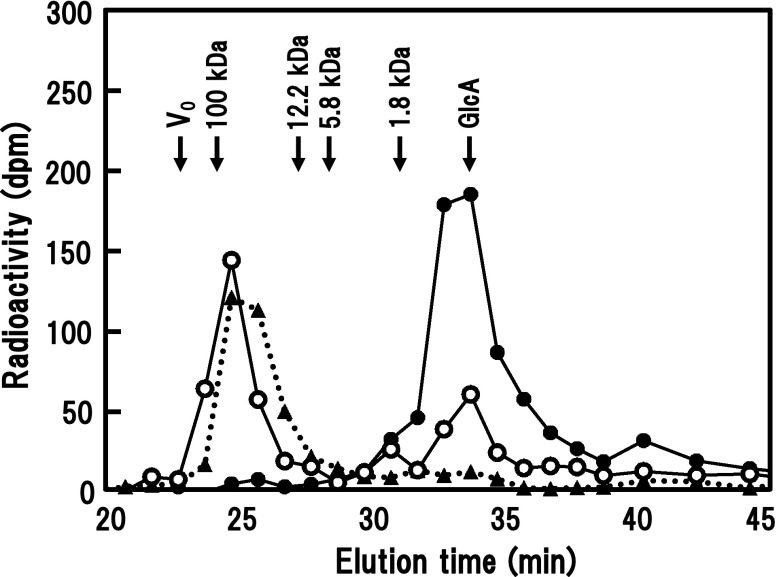
Analysis of the [^14^C]GlcA transfer product and its enzymatically degraded products by size-exclusion HPLC. Elution profile of the [^14^C]GlcA transfer product formed from a root AGP digested successively with α-l-arabinofuranosidase and endo-β-(1 → 6)-galactanase as the exogenous acceptor is shown. *Dotted lines* Intact transfer product, *open circles* digestion with β-glucuronidase, *closed circles* digestion with exo-β-(1 → 3)-galactanase and β-glucuronidase. The eluates were fractionated and their radioactivity was counted. The column system was calibrated with high-*M*_r_ dextran (*V*_o_) and pullulans with known *M*_r_, (Shodex Standard P-82; Showa Denko, Tokyo, Japan), and GlcA

The hydrolysis products were also analyzed by size-exclusion HPLC (Fig. 5). The intact radiolabeled transfer product formed from the enzymatically modified AGP emerged as a peak (*M*_r_ around 60,000) at the same point as the acceptor substrate (Okemoto et al. 2003). Digestion of the transfer product with β-glucuronidase liberated only small amounts of free [^14^C]GlcA and did not diminish the high-*M*_r_ transfer product. This had also been observed in paper chromatographic analyses. The suppression of hydrolysis is apparently caused by the limitation of access of the enzyme to uronosyl residues located at short β-(1 → 6)-galactosyl side chains in the modified AGP (Haque et al. 2005). In contrast, successive digestion of the transfer product with exo-β-(1 → 3)-galactanase and β-glucuronidase liberated most of [^14^C]GlcA as a free monosaccharide. Almost the same chromatographic profiles were observed for the transfer product formed from β-(1 → 3)-galactan. These results support the view of the transfer action of radish β-GlcATs on to acceptors that was given above.

### Analysis of transfer products formed from oligosaccharide acceptors

**Fig. 6:**
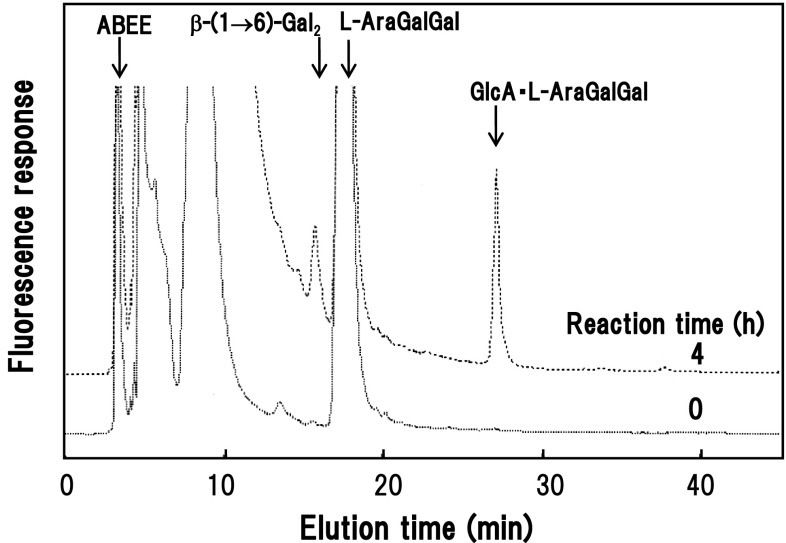
Formation of a GlcA transfer product form l-Ara·Gal·Gal as the acceptor substrate. The reaction was carried out at 25 °C for 4 h and products were analyzed by HPLC as ABEE derivatives. *Arrows* indicate the elution positions of standards and the transfer product

The GlcA transfer products with oligosaccharide acceptors were produced in a large-scale reaction with prolonged incubation time (24 h) and isolated as ABEE-derivatized compounds. Oligosaccharides subjected to β-GlcATs were β-(1 → 6)- and β-(1 → 3)-galactotrioses, and L-Ara·Gal·Gal (Table 3). For example, a GlcA transfer product was detected by HPLC when the enzymes were incubated with l-Ara·Gal·Gal as acceptor for 4 h (Fig. 6). Thus, the observed efficiencies of the oligosaccharides as acceptor substrates agreed with those calculated from the radiolabel method (Table 2). From β-(1 → 3)-galactotriose, two products I and II, possibly regioisomers, were produced. Transfer of single GlcA residues as β-anomers into each product was confirmed by sugar composition analysis, MALDI-TOF/MS, and enzymatic hydrolysis with β-glucuronidase and α-l-arabinofuranosidase. Digestion with the latter enzyme converted the product GlcA·L-Ara·Gal·Gal to GlcA·Gal·Gal, which coincided with standard β-GlcA-(1 → 6)-β-Gal-(1 → 6)-Gal by HPLC analysis, indicating the transfer of GlcA residues to either of the two Gal residues (Fig. 1).

**Table 3:** Analyses of GlcA transfer products formed from oligosaccharides

Acceptor^a^	Transfer product^b^	Elution time^c^ (min)	Transfer ratio^d^ (%)
β-(1 → 6)-galactotriose	GlcA·Gal_3_	35.0	1.8
β-(1 → 3)-galactotriose	GlcA·Gal_3_ I	31.0	2.2
	GlcA·Gal_3_ II	32.5	1.3
l-Ara·Gal·Gal	GlcA·L-Ara·Gal_2_	27.5	4.7

^a^The reaction was carried out at 25 °C for 4 h with 4 mM of oligosaccharide

^b^Compositions of the GlcA transfer products were confirmed by MALDI-TOF/MS, which yielded mass values of sodium adducts ([M+Na]^+^) consistent with calculated values for either [GlcA·Gal_3_-ABEE+Na]^+^ (*m/z* 852.8) or GlcA·L-Ara·Gal_2_-ABEE+Na]^+^ (*m/z* 822.7)

^c^Elution times of each product peak on the PALPAK Type *N* column are listed

^d^Transfer ratio is the percentage of GlcA transferred on to the acceptor calculated based on that of initial UDP-GlcA

**Fig. 7:**
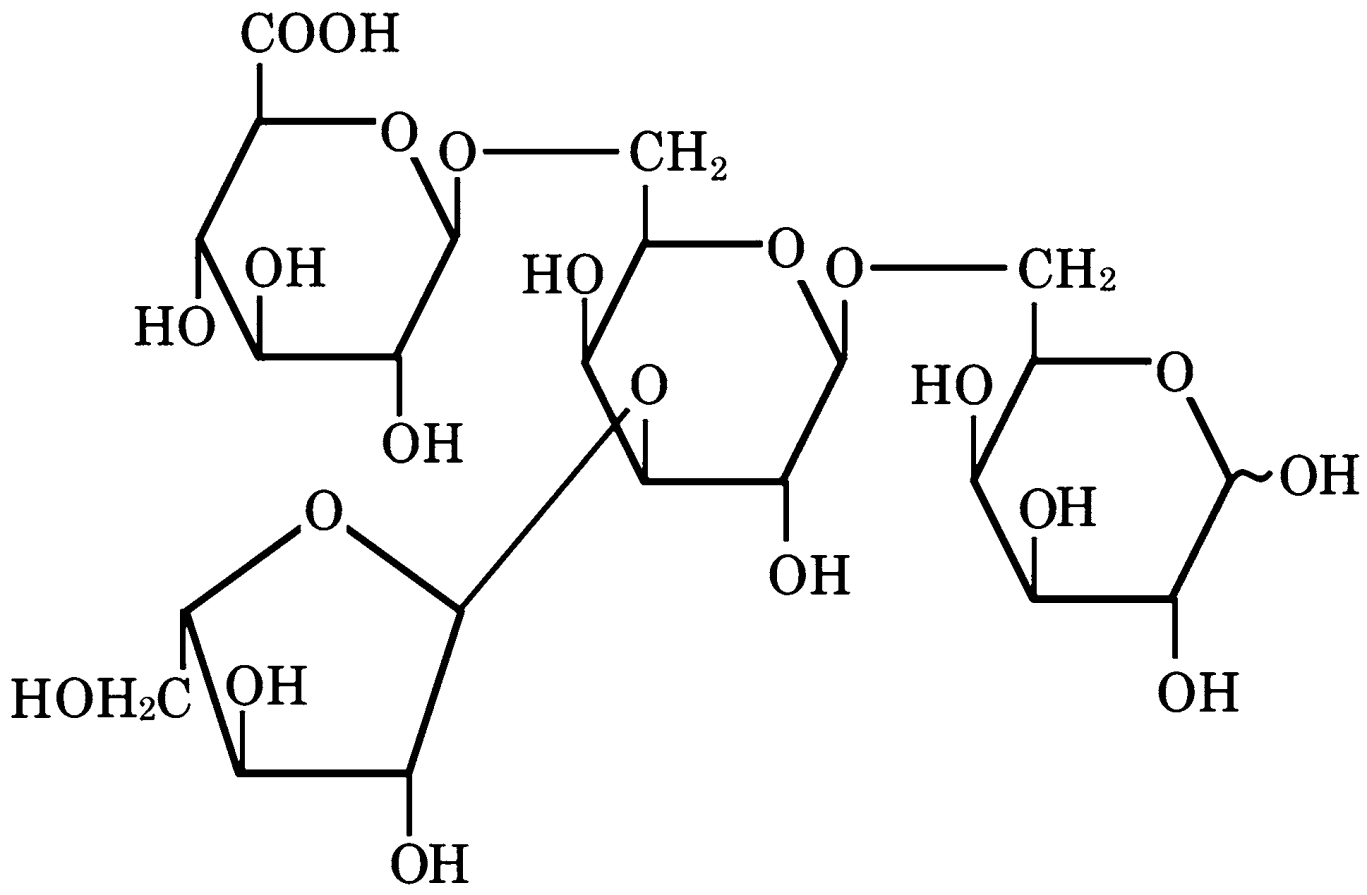
The structure of the GlcA transfer product GlcA·l-Ara·Gal·Gal

The structure of the transfer product GlcA·l-Ara·Gal·Gal was analyzed by ^1^H- and ^13^C-NMR and compared with the acceptor oligosaccharide, l-Ara·Gal·Gal (Table 4). The anomeric signals of the nonreducing terminal GlcA residues were observed at 4.46 (^1^H), and 102.6 ppm (^13^C). Signals due to C-6 and the methylene protons of its Gal′ residues in l-Ara·Gal·Gal at 61.1 ppm (^13^C), and 3.74 ppm (^1^H) shifted downfield to 69.1, and 3.85 and 4.00 ppm, respectively, for the transfer product in HC-HSQC NMR spectra (Supplemental Fig. S1). These data indicate that the GlcA groups were transferred on to C-6 of the Gal′ residues through β-linkages. Thus, the transfer product was identified as a branched tetrasaccharide, α-l-Araf-(1 → 3)[β-GlcA-(1 → 6)]-β-Gal-(1 → 6)-Gal (Fig. 7).

**Table 4:** NMR data and assignment of GlcA·L-AraGalGal-ABEE and L-AraGalGal-ABEE

	Chemical shift (ppm)							
	1	2	3	4	5	6	ABEE -CH_2_-	ABEE -CH_3_
GlcA·l-AraGalGal-ABEE								
^1^H signals								
GlcA	4.46	3.29						
l-Ara*f*	5.18	4.16	3.89	4.08	4.68			
					3.77			
Gal’	4.44		4.68	4.10		3.85		
						4.00		
Gal-ABEE	3.36					3.77	4.28	1.28
						3.98		
^13^C signals								
GlcA	102.6	73.1	75.7		73.7			
l-Ara*f*	109.3	81.4	76.7	83.9	61.2			
Gal′	103.2		80.1	67.9		69.1		
Gal-ABEE	45.8			76.0			61.7	13.7
l-AraGalGal-ABEE								
^1^H signals								
l-Ara*f*	5.19	4.16	3.90		3.79			
					3.70			
Gal′	4.42					3.74		
Gal-ABEE						3.78	4.28	1.28
						3.99		
^13^C signals								
l-Ara*f*	109.4	81.4	76.7	84.0	61.4			
Gal′	103.3		80.3	68.0		61.1		
Gal-ABEE	45.7			75.1		71.9	61.7	13.7

Values underlined are characteristic signals for identification of the GlcA transfer product, GlcA·l-AraGalGal, as ABEE derivative in comparison with the acceptor substrate, l-AraGalGal. Signals are not fully assigned due to limited amounts of the samples

Methylation followed by GLC analysis of the product gave terminal l-Ara*f* and GlcA (detected as Glc on GLC) residues, and *O*-3,6-linked Gal residues, supporting the above structure. However, the peak areas of these methylated sugars did not agree well with the expected equimolar ratio, possibly due to interference from ABEE in the analytical process. Based on similar analyses, the transfer product formed from β-(1 → 6)-galactotriose seems to be β-GlcA-(1 → 6)-β-Gal-(1 → 6)-β-Gal-(1 → 6)-Gal, and the regioisomer products I and II from β-(1 → 3)-galactotriose seem to be β-GlcA-(1 → 6)-β-Gal-(1 → 3)-β-Gal-(1 → 3)-Gal and β-Gal-(1 → 3)[β-GlcA-(1 → 6)]-β-Gal-(1 → 3)-Gal. These results confirm that radish GlcATs catalyze the transfer of GlcA residues from UDP-GlcA on to acceptors preferentially through β-(1 → 6) linkages.

### Effects of several nucleotide sugars on β-GlcAT reaction

Since we applied a membrane fraction, a crude enzyme preparation, from radish roots, it is apparent that the specimen contains many different glycosyltransferases involved in the synthesis of AG, besides β-GlcATs focused in this study. We examined the influence of several nucleotide sugars on the assay of activities of β-GlcATs using l-Ara·Gal·Gal as an acceptor substrate and several different nucleotide sugars (UDP-GlcA, -l-Ara, -Xyl, -Gal, -Glc, and GDP-Man, -Glc, and -l-Fuc) as donor substrates. The products formed by transfer of sugars were detected by HPLC as Fig. 6 (Supplemental Table S1). Coincident with our previous study, activities of GalTs and l-fucosyltransferase(s) higher than those of β-GlcATs were observed in the same radish microsomes (Misawa et al. 1996; Kato et al. 2003). Even the structures of four separated transfer products formed from UDP-Gal were not analyzed further, they might be single Gal residues attached to different positions of the acceptor substrate and/or two separate or consecutive Gal groups transferred to the substrate. These transfer products could form if oligosaccharides (see Tables 2, 3) other than l-Ara·Gal·Gal are applied as acceptor substrates. The structure of the product formed from UDP-Glc was also unclear. Therefore, our HPLC assay system is not specific in detecting β-GlcAT activities. However, most of elution times of these transfer products differed from that (24.4 min) for GlcA·l-Ara·Gal·Gal, indicating that GlcA transfer product was unambiguously estimated by the HPLC analysis if any small amounts of various nucleotide sugars were present in the membrane fraction. Hence, our method is valid to estimate the amounts of GlcA transfer product catalyzed by radish β-GlcATs.

## Discussion

Recent studies on the synthesis of plant cell wall polysaccharides have identified many glycosyltransferase enzymes and genes, thus improving our understanding of the mechanisms and regulation of cell wall polysaccharide synthesis. Among these well characterized glycosyltransferases can be found several α- and β-GlcAT enzymes. For example, in the synthesis of glucuronoxylan, Mortimer et al. (2010) have identified a putative α-GlcAT encoded by *GUX1* and *GUX2* in *Arabidopsis*, which catalyzes the transfer of GlcA residues on to β-(1 → 4)-xylan chains through α-(1 → 2) linkages. Zeng et al. (2010) have identified and characterized α-GlcAT in wheat. They also demonstrated that the enzyme protein is associated with xylosyl- and l-arabinosyltransferase proteins. The enzyme complex works in a cooperative manner and regulates the synthesis of repeating oligosaccharide structures in the polysaccharide.

Although AGPs are ubiquitous in plants, not much is known about the enzymology of AG biosynthesis, and only a few enzymes, such as β-(1 → 3)-GalT (Qu et al. 2008), GalT catalyzing the synthesis of β-(1 → 6)-galactan side chains (Geshi et al. 2013), and α-l-(1 → 2)-fucosyltransferase (Wu et al. 2010), have been identified and characterized. No β-GlcAT involved in AG synthesis has been examined for its properties or cloned. In this study, we undertook the biochemical characterization of radish GlcATs, aiming for purification of the enzyme protein and identification of the corresponding gene. A membranous β-GlcAT specimen prepared from primary roots of radish exhibited maximal activity around pH 6.0 and required Mn^2+^ and Triton X-100. These properties are similar to the pH range (pH 7.0) and requirement of divalent cations reported for α-GlcAT from wheat seedlings for the synthesis of glucuronoxylan (Zeng et al. 2008). They are also similar to the requirement of divalent cations and detergent for α-GlcAT from pea epicotyls (Waldron et al. 1989).

Radish GlcATs catalyzed the transfer of single GlcA residues from UDP-GlcA preferentially on to β-(1 → 3)-galactan and α-l-arabinofuranosidase- and β-(1 → 6)-galactanase-modified AGP through β-(1 → 6) linkages. We conclude that the enzyme transfers GlcA residues on to both β-(1 → 3)-galactan and β-(1 → 6)-galactosyl side chains, consistent with the proposed structure of radish and *Arabidopsis* AGPs, in which GlcA (and/or 4-Me-GlcA) residues are attached to β-(1 → 3)-galactan as single residue branches as well as to β-(1 → 6)-galactosyl side chains at nonreducing terminals (Tsumuraya et al. 1990; Tryfona et al. 2012). It is highly probable that the microsomes contain different β-GlcAT proteins, and we speculate that they act coordinately by transferring GlcA to β-(1 → 3)- and β-(1 → 6)-galactan chains through β-(1 → 6) linkages. Essentially the same mode of action of GlcA was observed for oligosaccharide acceptors. Among oligosaccharides tested, an l-Ara-containing oligosaccharide, α-l-Ara*f*-(1 → 3)-β-Gal-(1 → 6)-Gal (l-Ara·Gal·Gal), served as a much more effective acceptor than β-(1 → 6)-galactobiose, and formed α-l-Ara*f*-(1 → 3)[β-GlcA-(1 → 6)]-β-Gal-(1 → 6)-Gal as the transfer product. This was also observed in the case of polymer acceptors: once Smith-degraded product, carrying l-Ara*f* residues, was a better acceptor than twice Smith-degraded product, even though the former has a lower proportion of β-(1 → 3)-linked Gal residues (Table 1). These results suggest that the l-Aar*f* residues facilitate the transfer of GlcA residues by the enzymes, and indicate the possibility that in vivo radish GlcATs act in a cooperative manner with l-arabinosyltransferase and other related glycosyltransferases, a scheme that has been demonstrated for GlcAT involved in the synthesis of glucuronoarabinoxylan (Zeng et al. 2010). We believe that our study contributes to the understanding of the mechanisms and regulation of the synthesis of the AG portion of AGPs.

## Electronic supplementary material

Below is the link to the electronic supplementary material.

Supplementary material 1 (PDF 296 kb)

## Abbreviations

ABEE: *p*-Aminobenzoic acid ethyl ester
AGP: Arabinogalactan protein
l-Ara*f*: l-Arabinofuranose
l-Ara·Gal·Gal: α-l-Ara*f*-(1 → 3)-β-Gal-(1 → 6)-Gal
DP: Degree of polymerization
GLC: Gas–liquid chromatography
GlcA: Glucuronic acid
GlcAT: Glucuronosyltransferase
MALDI-TOF/MS: Matrix-assisted laser desorption/ionization time-of-flight mass spectrometry
4-Me-GlcA: 4-*O*-Methyl-glucuronic acid
GalT: β-Galactosyltransferase
