# Supplementary material for: Biosynthesis of the carbohydrate moieties of arabinogalactan proteins by membrane-bound β-glucuronosyltransferases from radish primary roots
Source: Planta. 2013 Sep 22;238(6):1157–69. doi: 10.1007/s00425-013-1959-0 (PMC3898515; doi:10.1007/s00425-013-1959-0)
Supplement: Supplementary file 1 — Supplementary material 1 (PDF 296 kb) [file 425_2013_1959_MOESM1_ESM.pdf]

Legend to Supplemental Figure

Fig. 1 ^{1}H-^{13}C HSQC NMR spectra of the GlcA transfer product, GlcA·L-AraGalGal-ABEE (red) and L-AraGalGal-ABEE (black). Signals for Gal' residues in L-AraGalGal at 61.1 ppm (^{13}C), and 3.74 ppm (^{1}H) shifted downfield to 69.1, and 3.85 and 4.00 ppm, respectively, indicating that the GlcA groups are transferred on C-6 of the Gal' residues.

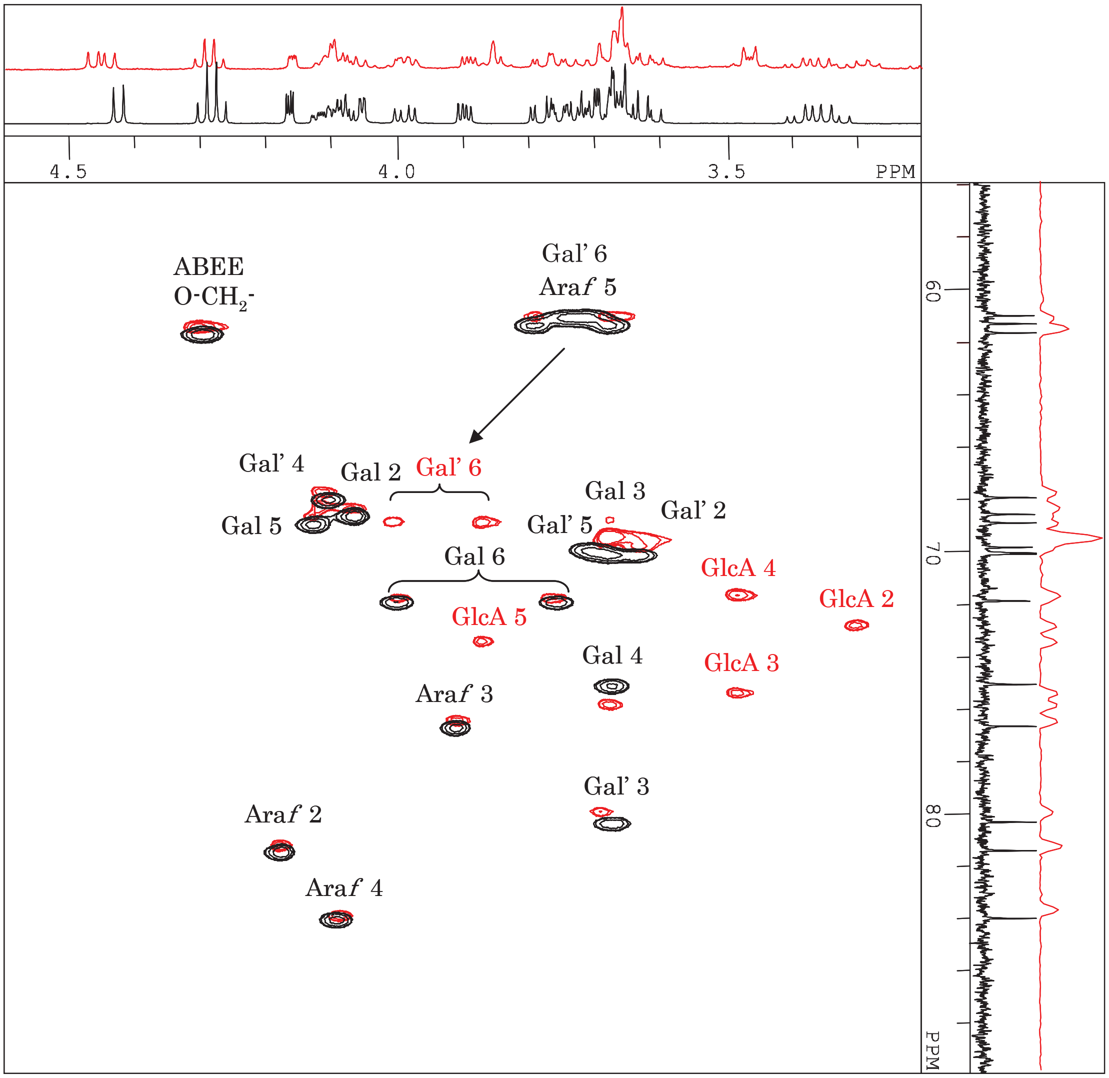

Endo et al., Supplemental Fig. 1

Supplemental Table 1 Transfer products formed from L-Ara·Gal·Gal as an acceptor substrate and various nucleotide sugars as donor substrates by the action of radish membranes.

Nucleotide sugar[a]	Elution time[b] (min)	Transfer ratio[d] (%)	Transfer product (pmol)	Specific activity [pmol min^{-1} (mg $protein)^{-1}$]
UDP-GlcA	24.4	3.07	3680	212.96
UDP-L-Ara	-[c]	0.00	0	0.00
UDP-Xyl	-	0.00	0	0.00
UDP-Gal	20.6	1.35	1625	94.04
	24.6	0.42	508	29.42
	38.4	1.65	1985	114.88
	47.4	1.42	1707	98.79
UDP-Glc	47.3	5.59	6710	388.28
GDP-Man	-	0.00	0	0.00
GDP-Glc	-	0.00	0	0.00
GDP-L-Fuc	21.1	10.09	12104	700.46
	26.8	0.21	249	14.40

Experiment was conducted as described for Fig. 6 by incubation of L-Ara·Gal·Gal as an acceptor substrate and various nucleotide sugars as donor substrates with the radish membrane fraction at 25 °C for 4 h. The reaction products were derivatized with ABEE, and estimated by HPLC.

[a]Nucleotide sugars were purchased from Sigma-Aldrich Japan except for UDP-L-Ara, which was synthesized enzymatically [Kotake et al., (2004) UDP-sugar pyrophosphorylase with broad substrate specificity toward various monosaccharide 1-phosphare from pea sprouts. J Biol Chem 279:45728-45736].

[b]Elution times of peaks formed by transfer of sugars on L-Ara·Gal·Gal. Since the HPLC instrument differed from that for Table 3, the elution time (24.4 min) for GlcA·L-Ara·Gal·Gal was somewhat earlier that that (27.5 min) in Table3.

[c]Not detectable.

[d]The percentage of sugars transferred on the acceptor calculated based on the initial nucleotide sugars (see Table 3).
